# Inappropriate Metacognitive Status Increases State Anxiety in Genetic Counseling Clients

**DOI:** 10.3389/fpsyg.2022.871416

**Published:** 2022-05-12

**Authors:** Yuka Shibata, Masaaki Matsushima, Megumi Takeuchi, Momoko Kato, Ichiro Yabe

**Affiliations:** ^1^Division of Clinical Genetics, Hokkaido University Hospital, Sapporo, Japan; ^2^Faculty of Medicine, Graduate School of Medicine, Department of Neurology, Hokkaido University, Sapporo, Japan

**Keywords:** metacognition, metacognitive theory, MCQ-30, state–trait anxiety, genetic counseling

## Abstract

**Background:**

Many genetic counseling (GC) studies have focused on anxiety status because clients of GC often feel anxious during their visits. Metacognition is known to be one of the causes of having an inappropriate thinking style. In this study, we examined the relationship between anxiety and the metacognitive status of GC clients according to their characteristics.

**Methods:**

The participants were 106 clients who attended their first GC session in our hospital from November 2018 to March 2021. The survey items were the clients’ characteristics, anxiety status at the time of the visit, and metacognitive status.

**Results:**

High state anxiety and high trait anxiety were observed in 34.9 and 11.3% of clients, respectively. Clients who were a relative or had a family history were significantly more likely to have high state anxiety. As for metacognitive status, only negative beliefs about thoughts concerning uncontrollability and danger were associated with having an anxiety status. Furthermore, multivariate analysis showed that negative beliefs about thoughts concerning uncontrollability and danger were an independent determinant of higher state anxiety, but not being a relative or having a family history. Metacognitive status scores were significantly lower in clients than in the control group.

**Conclusion:**

State anxiety was shown to be more dependent on negative beliefs about thoughts concerning uncontrollability and danger of GC clients than their characteristics such as being a relative or having a family history. The results of this study will contribute to the development of new GC psychosocial support measures to address the anxiety of GC clients.

## Introduction

Genetic counseling (GC) is the process to help people understand and adapt to the genetic contributions to disease *via* three approaches: situational interpretation, information provision, and psychosocial support (National Society of Genetic Counselor’s Definition Task Force et al., 2006). GC clients are not only patients with genetic diseases but are also their relatives, and various responses are required to meet the needs of clients with different backgrounds. It is expected that when a client undergoes psychosocial stress due to a life event, they will re-recognize the genetic risk, become aware of their emotional changes, and attend GC. Indeed, most GC clients with neuromuscular disease are reportedly aged in their 20s or 30s and are triggered to seek GC by life events, e.g., pregnancy or the desire to have a baby (Shibata et al., 2019). Whereas, since the type and degree of cognition of genetic risk and the degree of emotional change vary from person to person, it is important for a genetic counselor to have a good understanding of clients’ cognitive and emotional status. Clients often have fears and anxieties when they attend GC (Lewit-Mendes et al., 2018). It is important to be aware of the anxiety status of clients at an early stage, because a high level of anxiety may disrupt their thoughts and behaviors (Veach et al., 2003). For this reason, many studies have focused on the change in the anxiety status of clients, and it has been cited as one of the main outcome measures in GC (Madlensky et al., 2017). For instance, some studies that measured anxiety status before and after GC focused on clients’ characteristics (Pieterse et al., 2011), the approach for GC (Hunter et al., 2005), and comparisons with other cognitive status and emotions (Voorwinden et al., 2020). Conversely, only a few studies have examined what kind of thoughts clients have before receiving GC and what kind of anxiety they feel.

Metacognition is defined as “thinking about thinking” and is a high-order cognitive activity (Metcalfe and Shimamura, 1994). The term “metacognition” originated in the field of developmental psychology, and the concepts of “metacognitive knowledge” and “metacognitive activity” have since become popular (Brown, 1987; Fravell, 1987; Schraw and Moshman, 1995; Efklides, 2008). Drigas and Mitsea (2021a) proposed the 8 pillars × 8 layers model of metacognition, in which metacognition consists of eight components, such as learning theory, self-observation, and self-regulation, supported by eight stages of consciousness and intelligence (Drigas and Mitsea, 2021a). The prefrontal cortex, which is the region responsible for cognitive and executive functions, emotional control, and motivational functions, is closely related to metacognition. The medial and lateral prefrontal cortex and a network of regions including the precuneus and insula have been shown to be associated with metacognition, particularly the right dorsolateral prefrontal cortex, which is preferentially associated with metacognitive decision making (Vaccaro and Fleming, 2018). The prefrontal cortex is also reportedly associated with consciousness; however, there are various theoretical models of consciousness (Sattin et al., 2021), and there are reports describing metacognition as the vector of consciousness (Drigas and Mitsea, 2020). In addition, metacognition can control stress-related hormone secretion through sympathetic and parasympathetic mechanisms (Drigas and Mitsea, 2021b). From the above, metacognitive activity allows emotion and anxiety to be controlled. Whereas, prolonged mental and psychological disorders are considered to be influenced by inadequate thinking styles, e.g., continuous attention to anxious or fearful things, and metacognition can affect this thinking style (Wells and Cartwright-Hatton, 2004). Hence, the involvement of metacognition has been reported in patients with psychiatric disorders, e.g., generalized anxiety disorder and obsessive–compulsive disorder (Morrison and Wells, 2003; Sun et al., 2017). Metacognition is also correlated with social and health anxiety (Cartwright-Hatton and Wells, 1997), and studies have reported associations between metacognition and anxiety not only in psychiatric disorders but also in chronic diseases (Anderson et al., 2019; Capobianco et al., 2020; Lenzo et al., 2020), pain (Spada et al., 2016), epilepsy (Fisher and Noble, 2017), cancer survivors (Smith et al., 2018), caregivers of patients with neurological disorders (Siciliano et al., 2017), and parents of pediatric cancer patients (Toffalini et al., 2015). Metacognitive therapy aims to recognize and modify the inappropriate thinking styles of these subjects and has been shown to be effective in patients with mental illness (Sadeghi et al., 2015; Normann and Morina, 2018). As stated above, many GC studies have focused on anxiety status, but only a few have examined what kind of thinking styles increases the anxiety of clients. If the anxiety status of clients originates from their metacognitive status, it may be useful to examine the psychosocial support methods used in GC. Considering the above, we examined the relationship between the anxiety and metacognitive status of clients according to their characteristics.

## Materials and Methods

### Participants

The participants were individuals who attended their first GC session at our hospital between November 2018 and March 2021, and were aged ≥20 years at their first visit. The exclusion criteria were as follows: undergoing psychiatric treatment, possible cognitive decline due to primary disease, and visiting for prenatal chromosome testing without a family history. A control group was recruited to represent the general adult population to check metacognitive status. The inclusion criteria were males and females between the ages of 20 and 79 years and those who with a Mini Mental State Examination-Japanese score of <28 points were excluded because they may have mild cognitive impairment. For control group, all participants were recruited free of charge from the families of patients, staff members of our institution, and acquaintances of the researchers.

### Clinical Assessments

#### Client Characteristics

The survey items for the 10 characteristics [age, sex, patient or relative, pregnant (yes/no), married (yes/no), children (yes/no), family history (yes/no), type of disease, purpose of GC, and motivation to attend GC] were categorized based on the information in the medical records, referring to a previous survey (Shibata et al., 2019). For family history, if there was a second-degree relative who was genetically or clinically diagnosed with a hereditary disease or who met the test criteria for a hereditary disease, “family history” was used. If not, they were classified as “no family history.” The following diseases were observed in the subjects. Cancer: hereditary breast and ovarian cancer syndrome (*n* = 30), Lynch syndrome (*n* = 5), and familial adenomatous polyposis (*n* = 4); chromosomal disease: Down syndrome (*n* = 11), trisomy 18 (*n* = 5), and unbalanced translocation (*n* = 4); and neuromuscular disease: spinocerebellar ataxia (*n* = 4), Duchenne muscular dystrophy (*n* = 3), myotonic dystrophy type 1 (*n* = 3), and spinal muscular atrophy (*n* = 3).

#### Anxiety Status

Anxiety status was measured with the State–Trait Anxiety Inventory-Japanese version (STAI-JYZ), which is used in the clinical setting and its reliability and validity have been confirmed (Spielberger, 1983; Hidano et al., 2021). State anxiety is a transient situational response to an anxiety-producing event, i.e., “how one feels at a specific moment,” while trait anxiety is a stable response tendency to anxiety-producing events, i.e., “how one generally feels.” STAI-JYZ measures 20 items on a 4-point Likert-type scale; the higher the total score, the higher the state of anxiety. As males generally have higher average STAI-JYZ scores than females, the standard values were assessed separately for males and females (Table 1; Hidano et al., 2021).

**Table 1:** Stage table of STAI-JYZ.

State anxiety score	Level 5	Level 4	Level 3	Level 2	Level 1
Male	63 ~ 80	53 ~ 62	43 ~ 52	32 ~ 42	20 ~ 30
Female	64 ~ 80	52 ~ 63	41 ~ 51	30 ~ 40	20 ~ 29
**Trait Anxiety Score**					
Male	65 ~ 80	54 ~ 64	44 ~ 53	33 ~ 43	20 ~ 32
Female	66 ~ 80	54 ~ 65	43 ~ 53	32 ~ 42	20 ~ 31

In this study, the scores were examined separately between the two groups, instead of examining the results using continuous values, assuming that sex bias may affect the results. STAI-JYZ stages 1–3 were set as the low anxiety group, and stages 4 and 5 were set as the high anxiety group. Since trait anxiety is strongly correlated with metacognitive status (Cartwright-Hatton and Wells, 1997), only state anxiety was used as an assessment item for the relationship with metacognitive status in this study. The GC clients completed the STAI-JYZ before their first GC session.

#### Metacognitive Status

The Metacognitions questionnaire (MCQ) was designed to measure metacognitive beliefs and activities related to mental disorders, e.g., worry and intrusive thoughts (Cartwright-Hatton and Wells, 1997). In 2004, a shortened version of the MCQ (MCQ-30) was developed (Wells and Cartwright-Hatton, 2004) and in 2007, a Japanese version (MCQJ-30) was developed (Yamada and Tsuji, 2007). After obtaining the permission from the author who developed original MCQ-30 (Wells and Cartwright-Hatton, 2004), it was translated into Japanese (Yamada and Tsuji, 2007 and personal communication). The MCQ-30 measures 30 items using a 4-point Likert-type scale and assesses five independent subscales with six items each: positive beliefs about worry (Pos; e.g., “worrying helps me cope”); negative beliefs about thoughts concerning uncontrollability and danger (Neg; e.g., “when I start worrying, I cannot stop”); cognitive confidence (CC; e.g., “my memory can mislead me at times”); beliefs about the need to control thoughts (NC; e.g., “not being able to control my thoughts is a sign of weakness”); and cognitive self-consciousness (CSC; e.g., “I pay close attention to the way my mind works”). The reliability and validity of the MCQJ-30 have been confirmed for the general adult population (in submission). The GC clients completed the MCQJ-30 before their first GC session and control group did after consent to participate in the study.

### Statistical Analysis

Wilcoxon’s rank sum test was used to compare the MCQJ-30 score between GC clients and general adult individuals. The chi-square test and Fisher’s exact probability test were used to analyze the association between anxiety status and the characteristics of the clients, and logistic regression analysis was used to assess the association between the MCQJ-30 score and state anxiety status. Multiple logistic regression analysis was used to examine the association between the characteristics of the clients and MCQJ-30 score with state anxiety status. Cramer’s coefficient of association and correlation ratio were used to confirm multicollinearity. In all cases, a value of *p* <0.05 was treated as significant. JMP^®^ Pro ver. 15.2.0 (SAS Institute, Cary, NC, United States) was used for statistical analysis.

There were 12 participants (5.2%) in the GC client and control groups who had at least one item missing on the STAI-JYZ or MCQJ-30. The missing items were not common among individuals and were all different. In terms of age, one of these participants with missing data was in their 20s, one was in their 40s, four were in their 50s, three were in their 60s, and three were in their 70s, indicating that missing values were more likely to occur in the older age groups (*p* = 0.003). Therefore, we assumed that the missing values were caused by age (other observed data), i.e., missing at random, and we assigned the values using the least-squares method from the multivariate normal distribution of the non-missing parts.

### Ethical Considerations

Written consent to participate in this study was obtained from all subjects. This study was approved by the ethics committee of Hokkaido University Hospital.

## Results

### Participant Characteristics

There were 106 GC clients and 127 control participants. Mean age was 42.47 years in the GC client group and 49.59 years in the control group (*p* = 0.002). The proportion of females was 83% in the GC client group and 52% in the control group (Table 2). The characteristics of the GC client group are shown in Table 3. Patients accounted for 46% of clients, and those with a family history accounted for 76% of clients. Regarding disease type, cancer accounted for the largest number of clients (47%), followed by chromosomal (22%) and neuromuscular (20%) diseases. In terms of the purpose of GC, prenatal diagnosis was the most common (31%), followed by a definitive diagnosis (28%) and explanation of the disease (19%). The most common reason for attending GC was a life event of the client or their relatives (44%).

**Table 2:** Age and sex distribution of the GC client and control groups.

	GC client (*N* = 106)	Control (*N* = 127)	*Z*	Value of *p*
Age [years], mean (SD)	42.47 (13.58)	49.59 (16.91)	−3.16	0.002
20–29, *n* (%)	18 (17)	21 (17)		
30–39, *n* (%)	37 (35)	23 (18)		
40–49, *n* (%)	19 (18)	21 (17)		
50–59, *n* (%)	18 (17)	21 (17)		
60–69, n (%)	8 (8)	20 (16)		
70–79, *n* (%)	6 (6)	21 (17)		
Sex				
Male, *n* (%)	18 (17)	61 (48)		<0.001
Female, *n* (%)	88 (83)	66 (52)		

SD, Standard deviation; Z, Test statistics.

**Table 3:** Characteristics of the GC clients.

	*N* = 106
Age [years], *n* (%)	
20–29	18 (17)
30–39	37 (35)
40–49	19 (18)
50–59	18 (17)
60–69	8 (8)
70–79	6 (6)
Sex [female], *n* (%)	88 (83)
Patient [yes], *n* (%)	49 (46)
Pregnant [yes], *n* (%)	27 (25)
Married [yes], *n* (%)	84 (79)
Children [yes], *n* (%)	62 (58)
Family history [yes], *n* (%)	81 (76)
Type of disease	
Cancer, *n* (%)	50 (47)
Chromosomal disease, *n* (%)	24 (22)
Neuromuscular disease, *n* (%)	21 (20)
Cardiovascular disease, *n* (%)	5 (5)
Others, *n* (%)	6 (6)
Purpose of GC	
Disease explanation, *n* (%)	20 (19)
Definite diagnosis, *n* (%)	30 (28)
Explanation of test results, *n* (%)	5 (5)
Prenatal diagnosis, *n* (%)	33 (31)
Pre-symptomatic diagnosis, *n* (%)	16 (15)
Carrier diagnosis, *n* (%)	2 (2)
Motivation to attend GC	
Life event (including relative’s event), *n* (%)	47 (44)
Clinically suspected, *n* (%)	24 (23)
Just provided genetic diagnosis for probands, *n* (%)	16 (15)
Consultation from attending physician (secondary findings), *n* (%)	11 (10)
Others, *n* (%)	8 (8)

### Anxiety Status

In terms of state anxiety, 34.9% of clients were in the high anxiety group, which was slightly higher than reference (30%). The presence of state anxiety according to the characteristics of the clients is shown in Table 4. Clients who were relatives or who had a family history were significantly more likely to have high state anxiety (*p* = 0.013 and *p* = 0.030, respectively). There was also a tendency for life events to be a trigger for visits (*p* = 0.060). In terms of trait anxiety, 11.3% of clients were in the high anxiety group, which was less than reference (30%). The presence of trait anxiety according to the clients’ characteristics is shown in Table 5. There was no significant difference in any of the characteristics between the high and low trait anxiety groups.

**Table 4:** State anxiety by the characteristics of GC clients.

		High anxiety (*N* = 37)	Low anxiety (*N* = 69)	Value of *p*
Age [<40 years], *n* (%)		23 (42)	32 (58)	0.121
Sex [female], *n* (%)		34 (39)	54 (61)	0.104
Patient [yes], *n* (%)		11 (22)	38 (78)	0.013
Pregnant [yes], *n* (%)		10 (37)	17 (63)	0.788
Married [yes], *n* (%)		30 (36)	54 (64)	0.733
Children [yes], *n* (%)		20 (32)	42 (68)	0.497
Family history [yes], *n* (%)		33 (41)	48 (59)	0.030
Type of disease	Cancer [yes], *n* (%)	16 (32)	34 (68)	0.786
	Chromosomal disease [yes], *n* (%)	10 (42)	14 (58)	
	Neuromuscular disease [yes], *n* (%)	7 (33)	14 (67)	
	Cardiovascular disease [yes], *n* (%)	1 (20)	4 (80)	
	Others [yes], *n* (%)	3 (50)	3 (50)	
Purpose of GC	Disease explanation [yes], *n* (%)	4 (20)	16 (80)	0.155
	Definite diagnosis [yes], *n* (%)	8 (27)	22 (73)	
	Explanation of test results [yes], *n* (%)	2 (40)	3 (60)	
	Prenatal diagnosis [yes], *n* (%)	14 (42)	19 (58)	
	Pre-symptomatic diagnosis [yes], *n* (%)	7 (44)	9 (56)	
	Carrier diagnosis [yes], *n* (%)	0 (0)	2 (100)	
Motivation to attend GC	Life event (including relative’s event) [yes], *n* (%)	21 (45)	26 (55)	0.234
	Clinically suspected [yes], *n* (%)	6 (25)	18 (75)	
	Just provided genetic diagnosis for probands [yes], *n* (%)	6 (38)	10 (62)	
	Consultation from attending physician (secondary findings) [yes], *n* (%)	1 (9)	10 (91)	
	Others [yes], *n* (%)	1 (33)	2 (67)	

Percentages represent individuals with high and low anxiety in each population, e.g., in the population aged under 40 years, 42 and 58% of individuals had high and low anxiety, respectively.

**Table 5:** Trait anxiety by the characteristics of GC clients.

		High anxiety (*N* = 12)	Low anxiety (*N* = 94)	Value of *p*
Age [<40 years], *n* (%)		9 (16)	46 (84)	0.126
Sex [female], *n* (%)		11 (12)	77 (88)	0.686
Patient [yes], *n* (%)		4 (8)	45 (92)	0.377
Pregnant [yes], *n* (%)		3 (11)	24 (89)	1.000
Married [yes], *n* (%)		10 (12)	74 (88)	1.000
Children [yes], *n* (%)		6 (10)	56 (90)	0.526
Family history [yes], *n* (%)		10 (12)	72 (88)	0.727
Type of disease	Cancer [yes], *n* (%)	3 (6)	47 (94)	0.170
	Chromosomal disease [yes], *n* (%)	3 (12)	21 (88)	
	Neuromuscular disease [yes], *n* (%)	3 (14)	18 (86)	
	Cardiovascular disease [yes], *n* (%)	1 (20)	4 (80)	
	Others [yes], *n* (%)	2 (33)	4 (67)	
Purpose of GC	Disease explanation [yes], *n* (%)	1 (5)	19 (95)	0.455
	Definite diagnosis [yes], *n* (%)	4 (13)	26 (87)	
	Explanation of test results [yes], *n* (%)	0 (0)	5 (100)	
	Prenatal diagnosis [yes], *n* (%)	5 (15)	28 (85)	
	Pre-symptomatic diagnosis [yes], *n* (%)	1 (6)	15 (94)	
	Carrier diagnosis [yes], *n* (%)	3 (6)	47 (94)	
Motivation to attend GC	Life event (including relative’s event) [yes], *n* (%)	8 (17)	39 (83)	0.391
	Clinically suspected [yes], *n* (%)	2 (8)	22 (92)	
	Just provided genetic diagnosis for probands [yes], *n* (%)	1 (6)	15 (94)	
	Consultation from attending physician (secondary findings) [yes], *n* (%)	0 (0)	11 (100)	
	Others [yes], *n* (%)	1 (33)	2 (67)	

Percentages represent individuals with high and low anxiety in each population, e.g., in the population aged under 40 years, 16 and 84% of individuals had high and low anxiety, respectively.

### Metacognitive Status

The metacognitive status of the GC client and control groups is shown in Table 6. The mean total MCQJ-30 score was significantly lower in the GC client group (59.75) than in the control group (62.31; *p* = 0.015). Compared with the control group, the MCQJ-30 score of the GC client group was significantly lower only in the 30–39 years age group (*p* = 0.010). As for sex, females in the GC client group had a significantly lower MCQJ-30 score (*p* = 0.013). In each of the GC client and control groups, there was no significance difference in the total MCQJ-30 scores between age groups and between males and females (Supplementary Table 1). For each subscale, the Pos, CC, and CSC items were significantly lower in the GC client group than in control group (Supplementary Table 2). In the GC client group, there was no significant difference in the total MCQJ-30 score for any of the characteristics (Supplementary Table 3).

**Table 6:** Comparison of the total MCQJ-30 score by age group and sex using the Wilcoxon rank sum test.

	GC client (*N* = 106)	Control (*N* = 127)	*Z*	*r*	Value of *p*
Total, mean (SE)	59.75 (1.22)	62.31 (0.93)	−2.42	0.16	0.015
Age [years], mean (SE)					
20–29	65.61 (3.14)	63.71 (2.17)	0.58	0.09	0.563
30–39	57.73 (1.65)	65.26 (1.91)	2.56	0.33	0.011
40–49	59.11 (2.59)	61.95 (2.22)	−1.38	0.22	0.167
50–59	56.72 (2.46)	62.48 (2.50)	−1.93	0.31	0.053
60–69	64.75 (4.50)	55.65 (2.26)	1.30	0.25	0.194
70–79	59.00 (4.12)	64.19 (2.26)	−1.11	0.21	0.267
Sex, mean (SE)					
Male	57.61 (2.53)	61.07 (1.39)	−1.12	0.13	0.262
Female	60.18 (1.26)	63.45 (1.24)	2.47	0.20	0.013

r, Effect size; SE, Standard error; and Z, Test statistics.

### Association of MCQJ-30 Score With State Anxiety

When the effect of the total and subscale MCQJ-30 scores on state anxiety was examined, only Neg was indicated as a variable that influenced state anxiety (odds ratio: 1.255, *p* < 0.001; Table 7).

**Table 7:** Associations between the MCQJ-30 score and state anxiety using logistic regression analysis.

	*χ*^2^ (Value of *p*)	Pseudo-R2	Odds ratio	B estimate	SE	Value of *p*
Total	1.66 (0.198)	0.01	1.021	−0.021	0.016	0.200
Pos	0.82 (0.365)	0.01	1.059	−0.057	−0.057	0.366
Neg	13.63 (<0.001)	0.10	1.255	−0.227	0.068	<0.001
CC	0.45 (0.504)	0.00	0.965	0.035	0.053	0.507
NC	0.00002 (0.996)	0.00	1.000	0.000	0.059	0.996
CSC	0.68 (0.408)	0.01	1.049	−0.048	0.058	0.408

Pos, Positive beliefs about worry; Neg, Negative beliefs about thoughts concerning uncontrollability and danger; CC, Cognitive confidence; NC, Beliefs about the need to control thoughts; CSC, Cognitive self-consciousness; and SE, Standard error.

### Determinants of State Anxiety

The cases who were relatives or who had a family history were significantly more likely to have high anxiety status (Table 4). In addition, the higher the Neg score of the GC client group, the more likely they were to have high anxiety status (Table 7). Accordingly, binomial logistic regression analysis was conducted to analyze how much “patient or relative,” “with or without a family history,” and “Neg” as explanatory variables affected state anxiety. The correlation coefficients were 0.599 between “patient or relative” and “with or without a family history,” 0.037 between “patient or relative” and “Neg,” and 0.024 between “with or without a family history” and “Neg.” However, only Neg was an independent determinant of increased state anxiety (odds ratio: 1.25, *p* = 0.001; Table 8).

**Table 8:** Associations between the clients’ characteristics and MCQJ-30 score with state anxiety using multiple logistic regression analysis.

Explanatory variable	Odd ratio	95% Confidence intervals	B estimate	SE	Value of *p*
Relative	2.04	0.69–6.07	0.357	0.278	0.199
Family history	2.18	0.50–9.57	0.389	0.377	0.302
Neg score	1.25	1.09–1.43	0.223	0.223	0.001

Multivariable model: *χ*^2^ = 20.27, value of *p* < 0.001, Pseudo-R2: 0.15. Neg, Negative beliefs about thoughts concerning uncontrollability and danger; SE, Standard error.

## Discussion

This cross-sectional study is the first to focus on the relationship between the anxiety status and metacognitive status of GC clients. While many reports have focused on the anxiety status of GC clients, none have examined their metacognitive status. It was found that the anxiety status of clients was influenced by their metacognitive status regarding mental problems, suggesting that inventions aimed at improving metacognition may be effective for clients with high anxiety. Therefore, this study provides new insights into how to intervene in the anxiety status of GC clients.

### Anxiety Status of GC Clients

In terms of state anxiety, clients who were relatives of a patient or who had a family history were significantly more likely to be in the high anxiety group, and clients who came for GC because of an event in their lives or the life of a relative also tended to be in the high anxiety group.

According to a report comparing the anxiety status of a group of clients with that of the general population and cancer patients, clients have a higher anxiety status than the general population, similar to this study. Conversely, those who receive GC on the recommendation of their physicians have a significantly higher anxiety status before GC than those who go voluntarily (Nordin et al., 2011). However, in the present study, those who came for GC at their own request due to life events tended to have higher state anxiety, while those who came on the recommendation of their physician due to secondary findings did not. The degree of anxiety among those who came to the clinic on the advice of their physicians depends on how well their physicians explained the secondary findings and GC to them. It is possible that the increase in the number of occasions when comprehensive gene analysis is routinely performed and the increased awareness of physicians to secondary findings has led to the introduction of explanations at the pre-test stage, which has recently led to a reduction in anxiety among clients.

In addition, patients with suspected hereditary tumors have a significantly higher pre-GC anxiety status than their relatives (Ballatore et al., 2020). In the present study, the results were different, as state anxiety tended to be higher among those who were relatives or who had a family history of the disease. One of the reasons for these different results is that the previous study used the Hospital Anxiety and Depression Scale as an anxiety measurement scale to measure psychiatric symptoms in patients with physical illness, while we used the STAI-JYZ. Reports examining correlations between scores on the STAI and Hamilton Scale for Anxiety and neuroimaging data found that the anterior cingulate cortex is the best predictor of Hamilton Scale for Anxiety scores, whereas no significant correlations are found between the limbic system and STAI scores (Donzuso et al., 2014). The finding that each anxiety scale shows different associations with neuroimaging data suggests that these scales may reflect different aspects of anxiety. It has also been reported that state and trait anxiety have different structural-functional patterns (Saviola et al., 2020). There is no standardized measure for assessing the anxiety status of GC clients. In the future, it is necessary to examine what types of anxiety clients tend to have and to select the most appropriate ranking scale. Furthermore, another reason for this discrepancy may be that the present study included not only cancer but also other types of disease, e.g., neuromuscular and chromosomal diseases. Since the psychological impact of genetic testing reportedly varies by disease type (Oliveri et al., 2018), it is necessary to increase the sample size and examine each area in the future.

### Metacognitive Status of GC Clients

The metacognitive status of clients with regard to mental disorders, e.g., worry and intrusive thoughts, was significantly lower than that of the control group, and they tended to be significantly lower on three subscales: Pos, CC, and CSC. Although trait anxiety and the MCQ-30 score are positively correlated (Cartwright-Hatton and Wells, 1997) the fact that the trait anxiety of clients tended to be lower than that of the control group is also consistent with the result that the metacognitive status of clients was lower than that of the control group, which is consistent with the finding that the metacognitive status of clients was lower than that of the control group. There were no significant differences in scores between age groups or between males and females, as reported by Wells and Cartwright-Hatton (2004). The relationship between metacognitive status and temperament and character traits has been reported by Gawęda and Kokoszka (2014) by using the MCQ-30 and Cloninger’s Temperament and Character Inventory (Gawęda and Kokoszka, 2014). This report showed that the largest correlation was between CC and self-directedness (*r* = −0.44), suggesting that GC clients tend to be highly self-directed. While temperament is susceptible to genetic influences, character is susceptible to acquired environmental and learning influences (Cloninger, 1994). Gawęda and Kokoszka (2014) showed that metacognitive status was more closely related to character compared to temperament. In the present study, the metacognitive status of clients differed from that of the control group, suggesting that clients may have been influenced more by environmental factors than genetic factors. In the future, investigating the character traits of clients may help us to examine the environmental factors that lead them to seek GC. Conversely, there was no significant difference in the MCQ-30 scores according to the clients’ characteristics, indicating that clients’ metacognitive status is not affected by their characteristics. Therefore, the intervention on metacognitive status can be considered equally for all of the characteristics of clients.

### Association of Metacognitive Status With State Anxiety

In a systematic review of 13 studies on the association between metacognitive status and anxiety and depression in patients with physical diseases, Neg was found to be positively correlated with anxiety and depression in all evaluated patients (Parkinson’s disease, heart disease, cancer, stroke, epilepsy, multiple sclerosis, fibromyalgia, and diabetes). Conversely, four scales other than Neg showed different characteristics depending on disease type (Capobianco et al., 2020). It is possible that the relationship between subscales other than Neg and state anxiety may be clearer by increasing the sample size and examining the results separately according to disease type.

In the above systematic review, the Hospital Anxiety and Depression Scale was used as an anxiety measurement scale in 9 of 13 studies. Regarding the relationship between state anxiety and metacognitive status, studies have reported that Neg, CC, and NC are positively correlated with state anxiety (Spada et al., 2010; Gawęda and Kokoszka, 2014). In the present study, no correlation was found for any subscales except Neg. Since the previous studies were all conducted on students, it is possible that the results differed from those of the present study because of age.

As a result of the analysis of the relationship between state anxiety and the characteristics and metacognitive status of clients, only Neg was found to be an independent determinant that increased the state anxiety of clients, indicating that the state anxiety of clients depends more on Neg than on their characteristics. Although the background of GC clients is becoming more diverse due to the development of genetic technology, the results suggest that the state anxiety of GC clients does not depend on their characteristics and that the basic attitude of all clients toward state anxiety should be the same, which is useful for future clinical practice. Conversely, for Neg, interventions targeting metacognition may be effective.

### Limitations

In the present study, we examined 106 clients and classified them using 10 characteristics. As mentioned in the Introduction, the clients attending GC had a variety of backgrounds; therefore, we were unable to recruit a sufficient number of clients to further subdivide these items to assess anxiety and metacognitive status. Since the number of samples that can be obtained at a single institution is limited, it is necessary to increase the number of samples by considering multicenter collaborative research in the future.

## Conclusion

This study showed that the state anxiety of GC clients depended more on negative metacognitive beliefs about thoughts concerning uncontrollability and danger than their individual characteristics. It is useful to note that metacognitive interventions may be effective for clients with high anxiety, regardless of their characteristics. Furthermore, interventions targeting metacognition may have the potential to become a new method of psychosocial support in GC. The results of this study will contribute to the development of new psychosocial support methods for the anxiety of GC clients, and will also serve as basic data to consider what efforts are needed to improve genetic literacy in the future.

## Data Availability Statement

The original contributions presented in the study are included in the article/Supplementary Material, further inquiries can be directed to the corresponding author.

## Ethics Statement

The studies involving human participants were reviewed and approved by Ethical Review Board for Life Science and Medical Research, Hokkaido University Hospital. The patients/participants provided their written informed consent to participate in this study.

## Author Contributions

YS designed and executed the experiments and wrote the manuscript. MT and MK collected the data. MM was supervisor and edited the manuscript. IY administered this study with acquisition of the financial support and contributed to the final version of the manuscript. All authors agreed to be accountable for the content of the work.

## Funding

This study was funded by a grant from the Japan Agency for Medical Research and Development (to IY; 21ek0109527h0001) and the Grants-in Aid from the Research Committee of CNS Degenerative Diseases under Research on Measures for Intractable Diseases from the Ministry of Health, Labour and Welfare, Japan (to IY; 20FC1049).

## Conflict of Interest

The authors declare that the research was conducted in the absence of any commercial or financial relationships that could be construed as a potential conflict of interest.

## Publisher’s Note

All claims expressed in this article are solely those of the authors and do not necessarily represent those of their affiliated organizations, or those of the publisher, the editors and the reviewers. Any product that may be evaluated in this article, or claim that may be made by its manufacturer, is not guaranteed or endorsed by the publisher.

## Abbreviations

CC: Cognitive confidence
CSC: Cognitive self-consciousness
GC: Genetic counseling
MCQ: Metacognitions questionnaire
MCQ-30: Shortened version of the MCQ
MCQJ-30: Japanese version of MCQ-30
NC: Beliefs about need to control thoughts
Neg: Negative beliefs about thoughts concerning uncontrollability and danger
Pos: Positive beliefs about worry
STAI-JYZ: State-trait anxiety inventory-Japanese version
