# Supplementary material for: Inappropriate Metacognitive Status Increases State Anxiety in Genetic Counseling Clients
Source: Front Psychol. 2022 May 12;13:871416. doi: 10.3389/fpsyg.2022.871416 (PMC9133628; doi:10.3389/fpsyg.2022.871416)
Supplement: Supplementary file 1 [file Data_Sheet_1.docx]

Supplementary 1. Total MCQJ-30 score between age groups and sex

|  | **GC client (N=106)** | **p-value** | **Control (N=127)** | **p-value** |
| --- | --- | --- | --- | --- |
| Total, mean (SE) | 59.75 (1.22) |  | 62.31 (0.93) |  |
| Age [years], mean (SE) | | | | |
| 20-29 | 65.61 (3.14) | 0.494 | 63.71 (2.17) | 0.081 |
| 30-39 | 57.73 (1.65) |  | 65.26 (1.91) |  |
| 40-49 | 59.11 (2.59) |  | 61.95 (2.22) |  |
| 50-59 | 56.72 (2.46) |  | 62.48 (2.50) |  |
| 60-69 | 64.75 (4.50) |  | 55.65 (2.26) |  |
| 70-79 | 59.00 (4.12) |  | 64.19 (2.26) |  |
| Sex, mean (SE) | | | | |
| Male | 57.61 (2.53) | 0.775 | 61.07 (1.39) | 0.179 |
| Female | 60.18 (1.26) |  | 63.45 (1.24) |  |

SE: Standard error

Supplementary 2. MCQJ-30 subscale score

|  | **GC client (N=106)** | **Control (N=127)** | **p-value** |
| --- | --- | --- | --- |
| **Pos, mean (SE)** | **13.15 (0.32)** | **14.17 (0.31)** | **0.033** |
| Neg, mean (SE) | 11.01 (0.35) | 10.09 (0.24) | 0.067 |
| **CC, mean (SE)** | **11.29 (0.38)** | **12.29 (0.34)** | **0.031** |
| NC, mean (SE) | 11.25 (0.33) | 11.71 (0.25) | 0.086 |
| **CSC, mean (SE)** | **13.05 (0.34)** | **14.06 (0.31)** | **0.027** |

Pos: Positive beliefs about worry, Neg: Negative beliefs about thoughts concerning uncontrollability and danger, CC: Cognitive confidence, NC: Beliefs about the need to control thoughts, CSC: Cognitive self-consciousness

SE: Standard error

Supplementary 3. Total MCQJ-30 score by the characteristics of GC clients

|  | | **Yes** | **No** | **p-value** |
| --- | --- | --- | --- | --- |
| Age [<40 years], mean (SE) | | 60.3 (1.77) | 59.14 (1.68) | 0.488 |
| Sex [female], mean (SE) | | 60.18 (1.38) | 57.61 (2.44) | 0.775 |
| Patient, mean (SE) | | 60.69 (1.90) | 58.93 (1.59) | 0.673 |
| Pregnant, mean (SE) | | 60.70 (1.82) | 59.42 (1.52) | 0.291 |
| Married, mean (SE) | | 59.01 (1.37) | 62.55 (2.67) | 0.520 |
| Children, mean (SE) | | 58.23 (1.47) | 61.89 (2.07) | 0.297 |
| Family history, mean (SE) | | 59.48 (1.37) | 60.60 (2.71) | 0.861 |
| Type of disease, mean (SE) | Cancer | 59.34 (1.82) | 60.11 (1.65) | 0.494 |
|  | Chromosomal disease | 61.13 (2.20) | 59.34 (1.44) | 0.317 |
|  | Neuromuscular disease | 56.71 (3.09) | 60.49 (1.31) | 0.170 |
| Purpose of GC, mean (SE) | Disease explanation | 59.10 (2.93) | 59.90 (1.35) | 0.799 |
|  | Definite diagnosis | 58.97 (2.47) | 60.05 (1.40) | 0.459 |
|  | Prenatal diagnosis | 59.30 (1.87) | 59.95 (1.57) | 0.905 |
|  | Pre-symptomatic diagnosis | 61.13 (3.48) | 59.50 (1.30) | 0.665 |
| Motivation  to attend GC, mean (SE) | Life event  (Including relative's event) | 59.45 (1.59) | 59.98 (1.80) | 0.726 |
|  | Clinically suspected | 59.71 (2.95) | 59.76 (1.33) | 0.581 |
|  | Just provided genetic diagnosis for probands | 56.94 (3.58) | 60.24 (1.29) | 0.297 |
|  | Consultation from attending physician (Secondary findings) | 60.64 (4.23) | 59.64 (1.28) | 0.934 |

SE: Standard error
